# Effects of Age on Dual Task Walking Performance as Measured Using a Smartphone Application in Middle-Aged Adults

**DOI:** 10.1093/geroni/igab046.638

**Published:** 2021-12-17

**Authors:** Junhong Zhou, Gabriele Cattaneo, Wanting Yu, Jose Tormos, Lewis Lipsitz, David Bartres-Faz, Alvaro Pascual-Leone, Brad Manor

**Affiliations:** 1 Harvard Medical School/Hebrew SeniorLife, Roslindale, Massachusetts, United States; 2 Universitat Autònoma de Barcelona, Barcelona, Catalonia, Spain; 3 Hebrew SeniorLife, Boston, Massachusetts, United States; 4 University of Barcelona, Barcelona, Catalonia, Spain; 5 Harvard Medical School, Boston, Massachusetts, United States; 6 Hinda and Arthur Marcus Institute for Aging Research, Harvard Medical School, Boston, Massachusetts, United States

## Abstract

After the age of 65, one’s ability to walk while performing an additional cognitive task (i.e., dual-tasking) is predictive of both future falls and cognitive decline. However, while it is well-known that older adults exhibit diminished dual-task performance, the time course of age-related dual-task decline has not been established. We thus conducted an analysis of data collected within the ongoing Barcelona Brain Health Initiative, a prospective population-based study characterizing the determinants of brain health maintenance in middle-aged adults. Cognitively-unimpaired participants (n=655) aged 40-65 years without neuro-psychiatric disease completed laboratory-based trials of walking normally (single-task) and walking while performing a verbalized serial subtraction task (dual-task). A smartphone-based gait assessment application was used to capture data and derive both the mean stride time (ST) and stride time variability (STV, defined as the coefficient of variation about the mean stride time) of each trial. The dual-task costs (DTC) to each gait metric were obtained by calculating the percent change from single- to dual-task conditions. We categorized participants into five groups according to age (e.g. Group 1: 40-45 years; Group 5: 60-65 years). Age group did not have an effect on single-task gait outcomes (p>0.51). However, the oldest age group, as compared to each of the other groups, exhibited greater DTC to both ST and STV (p<0.03). These results indicate that dual-task walking performance in particular may begin to diminish in late middle age even in the absence of detectable cognitive issues, DTC may offer a sensitive metric to age-related change in cognitive function.

